# HIV/AIDS knowledge level, awareness of public health centers and related factors: a cross-sectional study among Brazilians in Japan

**DOI:** 10.1186/s12889-023-17308-w

**Published:** 2023-11-30

**Authors:** Shiho Nagai, Teruyo Kitahara, Katsuyuki Kito, Masahito Hitosugi

**Affiliations:** 1 Division of Occupational and Environmental Health, Department of Social Medicine, Shiga University of Medical Science, Seta Tsukinowa Cho, Otsu City, 520- 2192 Shiga Japan; 2 Department of Hematology, Shiga University of Medical Science, Seta Tsukinowa Cho, Otsu City, Shiga Japan; 3 Division of Legal Medicine, Department of Social Medicine, Shiga University of Medical Science, Seta Tsukinowa Cho, Otsu City, Shiga Japan

**Keywords:** HIV testing, Language barrier, Foreign residents, Brazilians, Public health center in Japan, Questionnaire survey

## Abstract

**Background:**

Accurate information is essential so that HIV infection can be detected in time for initiation of HIV/AIDS treatment. Immigrants are at high risk for delayed HIV testing and diagnosis, but foreign residents in Japan also seem to face barriers to accessing HIV/AIDS care. We aimed to assess their knowledge level of HIV/AIDS and awareness of public health centers in Japan (PHCs), and to explore factors related to these items.

**Methods:**

We conducted a cross-sectional study of Brazilians, the largest group of foreigners living in Shiga, using an anonymous, self-administered questionnaire survey in Brazilian Portuguese and Japanese via the Internet and mail. A multiple logistic regression analysis was used to examine the factors related to “Knowledge of HIV/AIDS” and “Awareness of PHCs”.

**Results:**

A total 182 Brazilians responded. More than half of them were beginners in Japanese. Most respondents were familiar with HIV/AIDS, but only 58% knew the existence of PHCs, and only 25% knew that HIV testing is available at PHCs free of charge and anonymously. A multiple logistic regression analysis showed that PHCs were less recognized by those with intermediate (odds ratio: 5.70, 95% confidence interval: 1.53–21.23) and beginner (odds ratio: 6.81, 95% confidence interval: 1.98–23.45) Japanese proficiency than by those with advanced.

**Conclusions:**

This survey revealed the knowledge level of HIV/AIDS and awareness of PHC among Brazilians in Shiga. Their lack of awareness of PHCs due to language barriers may lead to delays in HIV testing among them. Therefore, it is important for PHCs to disseminate information about medical services related to HIV/AIDS in Portuguese and plain Japanese to facilitate their access to HIV testing. However, PHC efforts alone are not enough. Medical interpreters who are familiar with Brazilian culture and customs, and the clinics that employ them, could help the Brazilian community and PHCs to overcome the language barrier and provide efficient and appropriate medical care to Brazilians. This would be one way to eliminate delays in HIV testing for Brazilians in Shiga.

## Background

In 2022, the estimated number of people living with HIV (PLHIV) worldwide had grown to 39.0 million, and 1.3 million people became newly infected with HIV [1]. A total of 630,000 people died from AIDS-related illnesses in 2022 [1].

Social vulnerability in the host country, where there are legal and administrative barriers to HIV testing [2] as well as cultural and linguistic barriers [3], increases the risk of HIV infection among migrants [4] and is likely to delay HIV diagnosis.

Globally, HIV prevalence is higher among migrants than in the host population [5], and migrants living with HIV in Organization for Economic Cooperation and Development (OECD) and other high-income countries are reported to account for an increasing proportion of new HIV diagnoses in these countries [6]. Migration forces people to adapt to foreign cultures, customs, and languages, and increases the risk of HIV infection as a result of limited access to health care, and it is now considered necessary to view the risk of HIV infection as a result of social, economic, political, or cultural factors, rather than as a result of individual behavior as it has been previously viewed [7].An online survey [8] among Brazilians showed that HIV knowledge was poor among younger participants, participants with lower income and education, and participants who had never been tested for HIV. Blair et al. also reported that HIV knowledge was associated with increased use of pre-exposure prophylaxis (PrEP) among Brazilian men who have sex with men (MSM) [9]. However, we have not found any studies on the knowledge level of HIV among Brazilians living in Japan.

HIV testing is available at public health centers in Japan (PHCs) free of charge and anonymously in Japan. Although Brazil also provides universal and free access to HIV/AIDS diagnosis and treatment, the HIV testing rate among MSM in Brazil is still low [10].

According to the Annual AIDS Occurrence Report 2019 [11], the number of new HIV cases per 100,000 population was 0.610 for Japanese and 0.105 for foreigners in Japan as a whole, while in Shiga the number was 0.354 for Japanese and 0.071 for foreigners. The number of new HIV cases in Shiga is slightly lower than that in Japan as a whole for both Japanese and foreigners. On the other hand, the number of new AIDS cases per 100,000 population in Shiga was 0.283 for Japanese and 0.141 for foreigners, compared to 0.230 for Japanese and 0.034 for foreigners in Japan as a whole. The number of new AIDS cases in Shiga is almost the same for Japanese as for Japan as a whole, but four times higher for foreigners. This suggests that foreigners infected with HIV in Shiga tend to be unaware of their HIV infection until they become full-blown AIDS. In Brazil, the proportion of PLHIV in the population aged 15–49 years was 0.5% [12], being about 10 times higher than the proportion in Japan (0.05%) [13]. In our search, we found no data on HIV infection rates or AIDS incidence among Brazilians living in Japan.

**Fig. 1:**
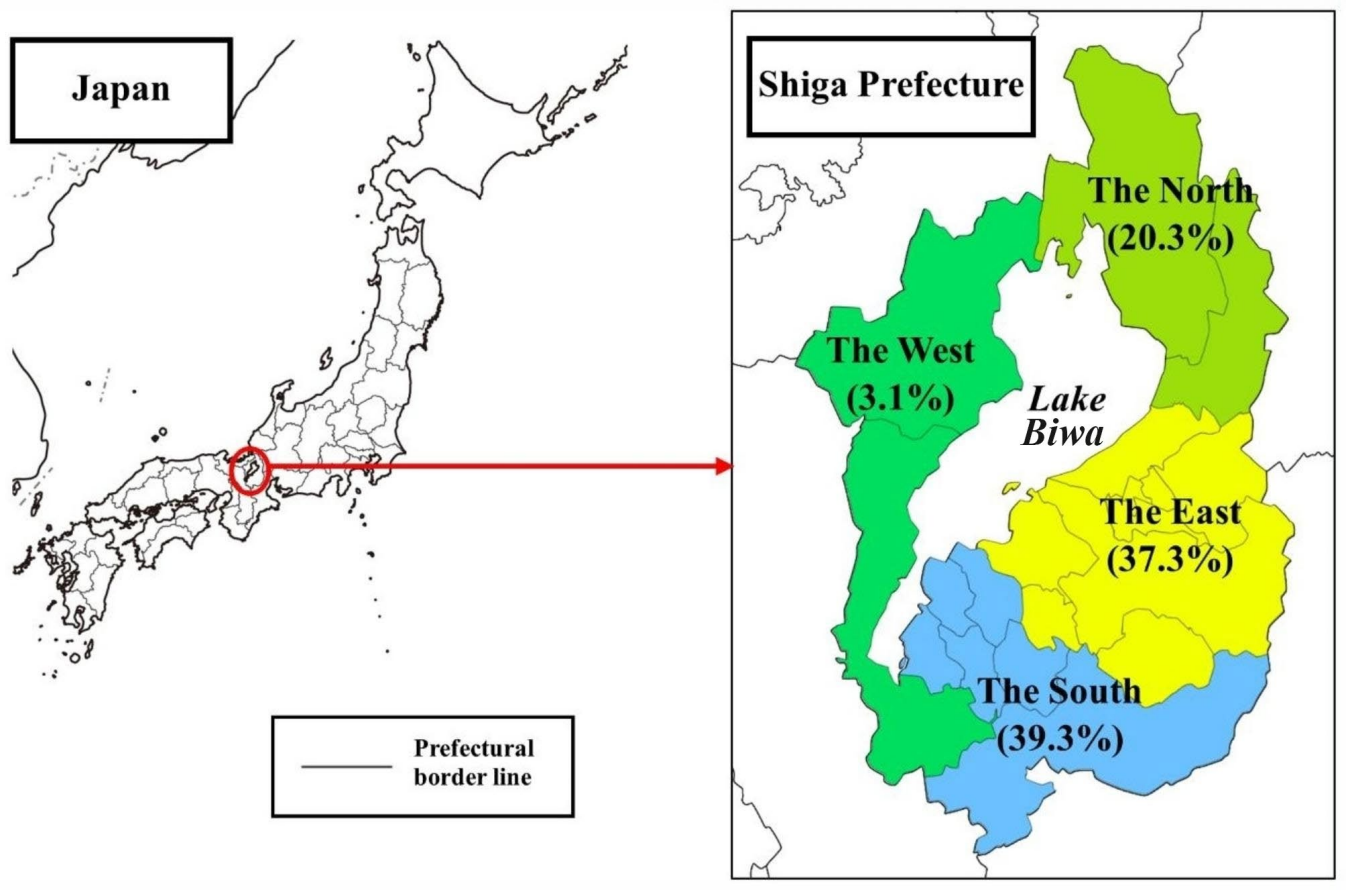
Map of Japan and Shiga Prefecture. Note: (); Percentage of Brazilian population distribution in the four regions of Shiga Prefecture

Shiga Prefecture is located almost in the center of Japan, and people live around Lake Biwa, the largest lake in Japan. There are several areas in Shiga with a high concentration of Brazilians, including descendants of former Japanese immigrants to Brazil [14] (Fig. 1). Many of them work in manufacturing factories, a major industry in Shiga [15]. In addition, the number of foreign workers in Shiga has been increasing in the past nine years, with Brazilians accounting for the largest number, approximately 30% [15]. It is estimated that there are approximately 30 Brazilian PLHIV aged 15–49 living in Shiga. However, only eight of the foreign PLHIV that were treated at Shiga University of Medical Science (SUMS) Hospital, the main provider of HIV/AIDS care in Shiga, have been Brazilian since it began providing HIV/AIDS care in 1997.

We were interested in how much knowledge Brazilians living in Shiga have about HIV/AIDS and what they do after acting at high risk of contracting HIV in Japan, where PrEP is not yet approved, unlike Brazil, where PrEP is approved [9]. Therefore, we aimed to assess their knowledge level of HIV/AIDS and the awareness of PHCs, and to explore factors related to these items in this study.

## Methods

### Study scenario

This cross-sectional study was conducted using a self-administered, anonymous questionnaire survey.

We first developed an original questionnaire referring to previous studies [16–19]. Then, we considered methods to deliver the questionnaire to as many of the survey targets as possible so that they would respond to it. The actual data sampling method will be described in detail later. The obtained responses were statistically analyzed at SUMS.

The data collection period, which was originally scheduled to run from April 1 to August 31, 2021, was extended to March 31, 2022 because the survey’s implementation was affected by the restriction of outings due to the increase in the number of COVID-19 patients.

### Response methods

The survey was conducted by combining a method of accessing and filling out a Google form via a QR code/URL included on a flyer (web survey method) and a method wherein completed questionnaires were to be directly returned to the university by mail (mail method). Respondents were instructed to choose one of the two methods to answer questions. For both the web and mail methods, a check box was provided at the beginning of the questionnaire to confirm the respondent’s consent.

### Data source

#### *Participants*

The survey population consisted of 7,539 Brazilian nationals (as of December 31, 2019), excluding those under 18 and over 80 years of age. The target sample size (n’) was calculated using the following Eq. 1 (assuming 5% margin of error, 95% confidence level, and 50% response rate) and corrected by the population size (7,539) using Eq. 2, resulting in 366 respondents.

1 $$n{\text{ }} = {\text{ }}{\lambda ^2}p\left( {1 - p} \right)/{d^2}$$

2 $$n'{\text{ }} = {\text{ }}nN/\left( {N + n - 1} \right)$$

n; target sample size before finite modification.

n’; target sample size.

λ; reliability (= 1.96).

d; allowable limit of error (= 0.05).

p; response rate (= 0.5).

N; population (= 7,539).

#### Data sampling

For data sampling, we selected 28 places that are frequently visited by Brazilians, and the first author visited them to solicit their cooperation in the survey. These places included one restaurant, six Brazilian fresh food stores, four city halls in Shiga, two Brazilian nursery schools, two Brazilian schools, two recruitment agencies, one Brazilian Portuguese-Japanese translation agency, three Catholic churches, one welfare council, four international associations, one Japanese language school, and one business office that directly employed Brazilians.

The first author requested a meeting with the person in charge at each site and visited the places that granted permission, explaining in detail the intent and purpose of this study. In cases where it was judged that it would be easier to communicate with the site representative in Brazilian Portuguese (hereafter referred to as Portuguese) than in Japanese, the first author either visited while accompanied by an interpreter or used an interpreter via telephone or ZOOM interview. A total of 25 interpreting requests were made to three professional interpreters. All 28 sites agreed to participate in the study.

The first author asked the cooperating individuals at each site to post flyers in their facilities describing the intent, purpose, and method of the survey, to distribute questionnaires and flyers to their customers and employees or co-workers, and to encourage those who received the flyers to respond to the questionnaire. The first author also personally visited the six Brazilian fresh food stores and three Catholic churches and asked customers and worshipers to respond to the survey. In addition, the Shiga International Association was asked to publish an article about this survey in its institutional newsletter, and on its website with a link to the webpage for responses. With the cooperation of interpreting consultants at the city halls, teachers and clerks at schools and nursery schools, and recruitment agencies, flyers for the survey and a call for responses were sent out via social networking sites that each group uses as their own personal contact network.

#### Original questionnaire items and language

Question items included respondents’ age, sex, sexuality, length of stay in Japan, existence of roommates (including family members), employment status, coverage of a Japanese public health insurance, existence of their family doctor/regular doctor, area of residence, primary language of daily life, ability to speak and read Japanese, knowledge of HIV/AIDS, and knowledge of the HIV/AIDS treatment system in Japan.

Respondents were asked to select one of the following five levels of their Japanese speaking ability. Level 1 (S1); can make speeches and debate, Level 2 (S2); can do simultaneous and conference interpretation, Level 3 (S3); can hold daily conversation, Level 4 (S4); can hold simple conversation, and Level 5 (S5); cannot speak it at all.

They were also asked to select one of the following five levels of their Japanese reading ability. Level 1 (R1); can understand newspaper editorials and reviews, Level 2 (R2); can understand general newspaper articles, Level 3 (R3); can understand an overview of information from newspaper headlines, Level 4 (R4); can read simple Japanese characters, and Level 5 (S5); cannot read it at all.

The above “speaking” and “reading” level classifications were established by the authors with reference to the Japanese Language Proficiency Test [20] and Japanese elementary school education standards. The best level of Japanese speaking/reading ability is S1/R1 and the most inadequate is S5/R5.

Referring to previous studies [16–19], we set up four questions to assess the knowledge level of HIV/AIDS, which were designed to inform respondents that recent HIV/AIDS treatment enables reintegration into society, motivates them to take HIV testing and treatment, encourages them to prevent secondary infection, and makes them aware that HIV/AIDS is not just someone else’s problem but something that affects them, their families, and their loved ones. The more correct answers to the four questions, the more correct knowledge about HIV/AIDS the respondent had.

Although many Brazilians speak Portuguese as their first language, there are some young Brazilians who have not fully determined whether their first language is Portuguese or Japanese because they were born in Japan or came to Japan with their parents when they were young and were therefore educated in Japan [21, 22]. Therefore, the language used for each questionnaire item was written in both Portuguese and Japanese. The Japanese was translated into Portuguese as simply as possible so that those with at least a secondary education could read and understand the content. Translation between Japanese and Portuguese was requested from the Center for Multicultural Society Kyoto, a non-profit organization.

### Analytical methods

The author loaded the responses from the web survey into Microsoft Excel, entered the mailed responses, and then tabulated the results by item.

A multiple logistic regression analysis was used to examine the factors related to “Knowledge of HIV/AIDS” and “Awareness of PHCs”. The dependent variable, “Knowledge of HIV/AIDS”, was binarized according to whether the respondents were aware of all four knowledge items (more knowledge) or three or fewer items (less knowledge). The dependent variable, “Awareness of PHCs”, was binarized according to the response to “Are you aware of the existence of PHCs?”.

For the independent variables to be included in the logistic regression analysis, we drew directed acyclic graphs (DAGs) [23] using the items of the basic attributes of the respondents in Table 1 and selected variables that were more strongly confounded by the two dependent variables. A DAG is a graph in which one-way arrows are used to represent known causal effects based on prior knowledge [23]. Regarding the number of independent variables, it was decided that there should be no more than 7 for each dependent variable, since there were 74 respondents for the question of knowledge level of HIV/AIDS for the smaller number and 76 respondents for the question of awareness of PHC for the smaller number.

**Table 1:** Basic attributes of respondents (n = 182)

Characteristics		Respondents	%
Sex	Female	98	53.8
	Male	84	46.2
Sexuality	Heterosexual	149	81.9
	Non-heterosexual	33	18.1
	Classification		
	Homosexual (Lesbian/Gay)	6	3.3
	Bisexual	4	2.2
	Do not want to answer/not sure	23	12.6
	Transgender	0	0.0
Age group(year)	18 ~ 29	27	14.8
	30 ~ 39	43	23.6
	40 ~ 49	56	30.8
	50 ~ 79	56	30.8
Length of stay in Japan(years)	Min	0	
	25%ile	5	
	50%ile	17	
	75%ile	25	
	Max	32	
Existence of roommates (including family members)	At least one roommate present	166	91.2
	Living alone	16	8.8
Employment Status	Employed^a^ or Self-employed^b^	151	83.0
	Student or unemployed	28	15.4
Coverage of a Japanese public health insurance	Covered	171	94.0
	Not covered	7	3.8
	No answer	4	2.2
Existence of their family doctor, or regular doctor	Yes	57	31.3
	No	121	66.5
	No answer	4	2.2
Area of residence	The South	58	31.9
	The East	61	33.5
	The North	62	34.1
	The West	0	0.0
Primary language of daily life	Japanese	13	7.1
	Non-Japanese	168	92.3
	Classification		
	Portuguese	159	87.4
	English	8	4.4
	Spanish	1	0.5

^a^ Employed: company employees, contract employees of public institutions, temporary employees, etc.

^b^ Self-employed: including agriculture

We finally selected independent variables as follows; “age”, “sex”, “existence of roommate(s)”, “employment status”, “length of stay”, and “Japanese proficiency”. “Age” was categorized into four groups of ≤ 29 years old, 30 years, 40 years, and 50 years or older, and “length of stay in Japan”, was categorized into four groups according to quartile values: ≤5 years, 6 to 17 years, 18 to 25 years, and ≥ 26 years. Japanese proficiency was reclassified into three groups: advanced (combination of [S1 + S2 + S3] and [R1 + R2 + R3]), beginner (combination of [S4 + S5] and [R4 + R5]), and intermediate (other than beginner and advanced).

The independent variables had a variance inflation factor of less than 1.6. All variables were entered using a forced entry method. The goodness of fit of the model was tested using the Hosmer-Lemeshow test. Data were analyzed using the Statistical Package for Social Sciences software program (SPSS, IBM version 29), and the significance level was set at 0.05.

## Results

### Respondent characteristics

There was a total of 182 respondents (62 from the web survey and 120 from the mail survey).

Table 1 shows the basic attributes of the respondents. The sex composition ratio was 53.8% female and 46.2% male. The sexuality composition was 81.9% heterosexual and 18.1% non-heterosexual. The 33 non-heterosexuals included 6 homosexuals (lesbian/gay), 4 bisexuals, and 23 who did not want to answer or did not know; there were no transgender individuals. Age was 18–29 years in 14.8%, 30–39 years in 23.6%, 40–49 years in 30.8%, and 50–79 years in 30.8%. The median length of stay in Japan was 17 years (minimum 0 years, maximum 32 years). 8.8% of respondents lived alone, and 15.4% of them were students or unemployed.

**Table 2:** Japanese proficiency of respondents (n = 182)

Characteristics	Respondents	%
Japanese speaking ability		
S1: Can make speeches and debate	9	4.9
S2: Can do simultaneous and conference interpretating	10	5.5
S3: Can hold daily conversations	52	28.6
S4: Can hold simple conversations	73	40.1
S5: Cannot speak it at all	35	19.2
Japanese reading ability		
R1: Can understand newspaper editorials and reviews	8	4.4
R2: Can understand general newspaper articles	11	6.0
R3: Can understand an overview of information from newspaper headlines	15	8.2
R4: Can read simple Japanese characters	100	54.9
R5: Cannot read it at all	43	23.6
Japanese proficiency		
Advanced^a^	32	17.6
Intermediate^b^	39	21.4
Beginner^c^	104	57.1

^a^ Advanced: (S1 + S2 + S3) and (R1 + R2 + R3)

^b^ Intermediate: except advanced and beginner

^c^ Beginner: (S4 + S5) and (R4 + R5)

The primary language of life was Japanese for 7.1% with Portuguese being the most common non-Japanese language at 87.4%. In Table 2, Japanese proficiency was 17.6% advanced, 21.4% intermediate, and 57.1% beginner.

### Knowledge of HIV/AIDS and awareness of the HIV/AIDS treatment system in Japan

Table 3 shows the respondents’ knowledge of HIV/AIDS. 69.8% of them knew that “there is a risk of infecting others even in the asymptomatic stage before the onset of AIDS”, 76.9% knew that “treatment for HIV/AIDS should be lifelong”, 79.1% knew that “PLHIV can live long lives just like non-PLHIV with proper treatment”, and 88.5% knew that “anyone can get HIV/AIDS”. 57.1% knew all four of the above facts. 36.4% of them answered that they had not received any information about HIV/AIDS in Japan.

**Table 3:** Respondents’ knowledge of Human immunodeficiency virus/ Acquired Immune Deficiency Syndrome (HIV/AIDS) (n = 182)

Questions	Answers	Respondents	%
“Do you know that there is a risk of infecting others even in the asymptomatic stage before the onset of AIDS?”	Yes	127	69.8
	No	53	29.1
	No answer	2	1.1
“Do you know that treatment for HIV/AIDS should be lifelong?”	Yes	140	76.9
	No	41	22.5
	No answer	1	0.5
“Do you know that with appropriate treatment, HIV-infected people can live as long as non-HIV infected people?”	Yes	144	79.1
	No	36	19.8
	No answer	2	1.1
“Do you know that anyone can get HIV/AIDS?”	Yes	161	88.5
	No	17	9.3
	No answer	4	2.2
Knowledge level of HIV/AIDS			
All 4 questions are Yes		104	57.1
Less than 3 questions are Yes		74	40.7
Not getting any information about HIV/AIDS in Japan		63	34.6

Table 4 shows the respondents’ awareness of the HIV/AIDS treatment system in Japan. 57.7% of them knew that “there are PHCs”, 24.7% knew that “HIV testing is available at PHCs free of charge and anonymously”, and 20.9% knew both of these facts. A total of 16.5% knew that “Japan has a medical subsidy system for receiving treatment for HIV/AIDS”, and 12.6% knew that “in Japan, telephone counseling is available for HIV/AIDS, patient support, and family support in Portuguese and Spanish”.

**Table 4:** Respondents’ knowledge of the HIV/AIDS treatment system in Japan (n = 182)

Questions	Answers	Respondents	%
“Are you aware of the existence of PHCs? “	YES	105	57.7
	NO	76	41.8
	No answer	1	0.5
“Do you know that HIV testing is available at PHCs free of charge and anonymously?“	YES	40	24.7
	NO	141	75.3
	No answer	1	0.5
Awareness of both PHCs and their work on HIV testing	Both questions are YES	38	20.9
	Only 1 question is YES	69	37.9
	Both questions are NO	75	41.2
“Do you know that Japan has a medical subsidy system for receiving treatment for HIV/AIDS?”	YES	30	16.5
	NO	149	81.9
	No answer	3	1.6
“Do you know that telephone counseling is available for HIV/AIDS, patient support, and family support in Portuguese and Spanish in Japan?“	YES	23	12.6
	NO	156	85.7
	No answer	3	1.6

^a^ PHCs: public health centers in Japan

### Factors related to “knowledge level of HIV/AIDS” and “awareness of PHCs”

The results of the multiple logistic regression analysis are presented in Table 5. The knowledge level of HIV/AIDS was lower among men (odds ratio [OR]: 3.05, 95% confidence interval [CI]: 1.46–6.37) than among women, and among the unemployed (OR: 6.41, 95% CI: 2.27–18.13) than among the employed. Regarding PHCs, those with intermediate (OR: 5.70, 95% CI: 1.53–21.23) and beginner (OR: 6.81, 95% CI: 1.98–23.45) for Japanese proficiency were less aware of the centers than advanced, and those living alone (OR: 3.93, 95% CI: 1.07–14.46) were less aware than those living with someone else.

**Table 5:** Factors associated with knowledge of HIV/AIDS and of PHCs^a^

	Having less amount of knowledge of HIV infection/AIDS^g^					Not having knowledge of PHCs ^h^		
Variables	OR^i^	95%CI^j^		p value		OR	95%CI	p value
Sex								
Female	Ref.					Ref.		
Male	**3.05**	**1.46–6.37**	**0.003**			1.92	0.95–3.88	0.068
Age group (year)								
~ 29	1.37	0.43–4.38	0.599			1.76	0.52–5.94	0.364
30 ~ 39	0.87	0.30–2.47	0.787			2.29	0.79–6.62	0.127
40 ~ 49	0.89	0.36–2.15	0.788			1.02	0.41–2.56	0.960
50 ~	Ref.					Ref.		
Length of stay (years)								
~ 5	0.61	0.17–2.17	0.446			2.23	0.67–7.50	0.194
6 ~ 17	1.35	0.40–4.61	0.628			0.97	0.29–3.30	0.964
18 ~ 25	2.37	0.83–6.75	0.108			1.14	0.38–3.45	0.816
26~	Ref.					Ref.		
Existence of roommates								
At least one roommate present	Ref.					Ref.		
Living alone	0.55	0.16–1.93	0.353			**3.93**	**1.07–14.46**	**0.040**
Employment Status								
Employed^b^ or Self-employed^c^	Ref.					Ref.		
Student or unemployed	**6.41**	**2.27–18.13**	**< 0.001**			0.54	0.19–1.55	0.254
Japanese proficiency								
Advanced^d^	Ref.					Ref.		
Intermediate^e^	0.57	0.19–1.76	0.327			**5.70**	**1.53–21.23**	**0.010**
Beginner^f^	0.80	0.29–2.21	0.665			**6.81**	**1.98–23.45**	**0.002**

^a^ PHCs: public health centers in Japan

^b^ Employed: company employees, contract employees of public institutions, temporary employees, etc.

^c^ Self-employed: including agriculture

^d^ Advanced: (S1 + S2 + S3) and (R1 + R2 + R3)

^e^ Intermediate: except advanced and beginner

^f^ Beginner: (S4 + S5) and (R4 + R5)

^g^ See Table 3. Less than 3 Yes to 4 questions concerning knowledge of HIV/AIDS.

^h^ See Table 4. The answer to the question, “Are you aware of the existence of public health centers in Japan (PHCs)?” was No.

^i^ OR: odds ratio

^j^ 95% CI: 95% Confidence Interval

## Discussion

This questionnaire survey revealed the following. The main language spoken by the respondents was Portuguese, and more than half of them were beginners in Japanese. The percentage of respondents who knew each knowledge items of HIV/AIDS was 70–89%, showing that more respondents had relatively new knowledge items. The percentage of respondents who knew all four knowledge items was 57%. At most, 58% of respondents were aware of PHC, and only 25% of the respondents knew that “HIV testing is available at PHCs, ‘free of charge’ and ‘anonymously’”. Multivariate analysis revealed that “sex” and “employment status” were related to the knowledge level of HIV/AIDS, while “Japanese language proficiency” and “having roommates” were related to the awareness of PHC.

Regarding knowledge of HIV/AIDS, the 2017 survey in Japan [24] found that 52.1% of Japanese had the impression that AIDS was a “deadly disease” and 33.6% believed that “the cause is unknown and there is no cure”, indicating that a considerable number of Japanese still had outdated knowledge of HIV/AIDS. The higher percentage of those with correct knowledge about HIV/AIDS in this survey may be due the fact that many of them acquired knowledge before coming to Japan. However, young Brazilians who come to Japan to work or Brazilian students living in Japan may have insufficient knowledge about HIV/AIDS, as it has been reported that people of younger age and with less education have less knowledge about HIV/AIDS and are less likely to be tested for HIV [8, 10, 25, 26]. Therefore, Japanese health care providers should make efforts to provide adequate HIV/AIDS knowledge to Brazilians in Japan.

The result of the multiple logistic regression analysis showed that men had less knowledge about HIV/AIDS than women, possibly because women are advised to be tested for HIV during pregnancy to prevent mother-to-child transmission of HIV [27]. Thus, there may be differences between men and women in their opportunities to get information about HIV/AIDS in Brazil. The results also showed that unemployed people had less knowledge about HIV/AIDS than those who were employed. Previous studies [8, 17] have also shown that employment status is one of the most important determinants of comprehensive HIV knowledge. To disseminate information about HIV/AIDS to men, unemployed persons, and students, it is necessary to find ways to disseminate this information, e.g., by posting information at workplaces, social spaces, public facilities (such as train stations and employment offices) and schools, which are places that these individuals are sure to visit.

Although the present survey would have a selection bias in that people with concerns about HIV/AIDS or Japanese proficiency were more likely to respond, the level of awareness of PHCs was not very high. While the percentage of Japanese who were aware of the availability of free and anonymous HIV testing at PHCs was relatively low at 52.0% [24], the percentage was even lower among the respondents in the present survey. Our analysis showed that respondents with intermediate or beginner Japanese proficiency were less familiar with PHCs than those with advanced. The author also found that the information on HIV testing provided by Shiga was detailed in Japanese but limited in Portuguese. To increase awareness of PHCs and promote HIV testing, it is necessary to disseminate information in “Yasashii Japanese [28]” and Portuguese, which is the primary language of this population, taking into account cultural competence and ethnic diversity [29, 30]. “Yasashii” is a Japanese word meaning “easy/kind/gentle/soft”, and “Yasashii Japanese” is a form of presentation that uses simple vocabulary and grammar and is intended for people with a beginner to intermediate level of Japanese proficiency. Another factor related to the perception of PHCs was the absence of people living together. It is necessary to provide information about PHCs to “Brazilians living alone” since previous studies [31, 32] also reported a high rate of HIV infection among “people living alone”.

According to Levesque et al. [33], who defined access to health care as “the opportunity to seek and obtain appropriate health care services, when the need for care is recognized”, the process can be divided into six steps: generating of health care needs, recognizing and wanting for health care, searching for health care services, reaching services, using services, and obtaining outcomes. To achieve these steps, the service providers must meet five criteria: “Approachability”, “Acceptability”, “Availability and Accommodation”, “Affordability”, and “Appropriateness”. We replaced the service users with Brazilians living in Shiga and the service providers with PHCs to examine at what stage of the process they could not reach PHCs. “Affordability” and “Appropriateness” in PHCs services are ensured by providing free HIV testing at PHCs and referral to specialized medical facilities if the test is HIV positive, respectively. However, at present in Shiga, PHCs as service providers are deficient in disseminating information about their free and anonymous HIV testing as well as outreach activities, which hinders “Approachability” to PHCs. It is also unclear whether PHCs have Portuguese medical interpreters, whether they can accommodate Portuguese-speaking Brazilians, and whether they can adequately protect the privacy of visitors. These issues hinder “Acceptability”. In addition, many foreign residents in Shiga are employed on a part-time basis, which makes it difficult for them to take time off from work, and HIV testing (available by appointment only) at all of seven PHCs in Shiga is offered only on limited days and for limited hours on weekdays, which also hinders the “Availability and Accommodation”. Even if they have knowledge about HIV/AIDS and can decide to undergo the HIV testing, the three aspects of “Approachability”, “Acceptability”, and “Availability and Accommodation” hinder them from acting on this knowledge, thereby making it difficult for them to access PHCs.

This Levesque et al. model of access to health care [33] assumes that there is little or no prejudice, discrimination, or stigma against a disease or its patients. In this light, the barrier to “accessibility” at the initial stage of treatment participation is likely to be higher for HIV/AIDS, where prejudice, discrimination, and stigma affect many service users, than for other diseases [9]. To improve the first aspect, “approachability”, it is necessary to remove the language barrier for service users, taking into account cultural and linguistic diversity, as pointed out in previous studies [29, 30].

Therefore, we propose a new intermediary between the Brazilian community in Shiga and the PHCs to interpret and explain the demands and explanations of both sides and to provide efficient and appropriate medical care to Brazilians beyond the language barrier. For example, an ideal intermediary could be a well-trained medical interpreter who is familiar with the Brazilian culture and customs, or a clinic that hires such interpreters. We also propose the other thing to add information about PHCs in the “Guidebook for Living and Working in Japan - For Foreigners Starting Life in Japan” [34] distributed by the Immigration Bureau to foreign residents who decide to live or work in Japan.

A lack of awareness of PHCs due to the language barrier may lead to delays in HIV testing among Brazilians in Japan. It is important for PHCs to disseminate information on medical services related to HIV/AIDS in both Portuguese and “Yasashii Japanese [28]” and to create a system to facilitate HIV testing for informal workers to receive. This will benefit not only Brazilians but also Japanese people.

Our survey method had many difficulties and needed considerable time and effort to get respondents, so it was only able to reach about half of our target sample size, which was a limitation of the present survey method. Since the COVID-19 pandemic kept many people from going out, the first author and those responsible for the cooperating sites had lost many opportunities to inform participants about this questionnaire. In addition, although the questionnaire was prepared in simple Japanese and Portuguese, we speculate that many participants may have stopped answering the questionnaire halfway through because it appeared complicated and the topics were difficult to understand because it was written in both languages. However, we determined that we had sufficient responses for statistical examination, as the regression model could be constructed with all items considered to be confounding factors in our analysis. The representativeness of the respondents was examined in terms of sex, age, and area of residence. According to the Statistics of Foreign Residents in Shiga [35], the age distribution of Brazilians was 18.8% 18–29 years old, 26.2% 30–39 years old, 24.5% 40–49 years old, and 30.5% 50–79 years old. The sex composition was 45.2% female and 54.8% male. According to the Basic Resident Registration Population Survey [14] (see Fig. 1), the number of Brazilians living in Shiga in the west was originally smaller than in other areas. The results of the present survey are not so different from those statistics, showing that the responses are unbiased. Finally, a limitation of this survey is that the results were obtained from a specific region of Japan, making it difficult to generalize them to Japan as a whole.

## Conclusions

The present survey revealed the level of HIV/AIDS knowledge and awareness of PHCs among Brazilians living in Shiga. That is, they did not much aware of PHCs while they had the knowledge of HIV/AIDS. Sex and employment status were found to play roles in the knowledge level of HIV/AIDS, while having a roommate and Japanese language proficiency played roles in the level of awareness of PHC.

We recognized that there is a need to develop educational interventions for HIV/AIDS prevention for service users that are sensitive to cultural background and ethnic diversity, and to continue to develop access available for prevention in today’s health care system.

## Data Availability

The data that support the findings of this study are available from the corresponding author upon reasonable request.

## List of abbreviations

*HIV*: Human immunodeficiency virus
*AIDS*: Acquired immunodeficiency syndrome
*HIV/AIDS*: Human immunodeficiency virus/Acquired Immune Deficiency Syndrome
*PHCs*: Public health centers in Japan
*PLHIV*: People living with HIV
*SUMS Hospital*: Shiga University of Medical Science Hospital
*OECD*: Organization for Economic Cooperation and Development
*PrEP*: pre-exposure prophylaxis
*MSM*: men who have sex with men
*DAGs*: directed acyclic graphs
*OR*: Odds ratio
*CI*: Confidence interval
